# 惰性B细胞淋巴瘤诊断与鉴别诊断中国专家共识（2025年版）

**DOI:** 10.3760/cma.j.cn121090-20250220-00081

**Published:** 2025-07

**Authors:** 中国抗癌协会血液肿瘤专业委员会 中华医学会血液学分会淋巴细胞疾病学组 中国惰性淋巴瘤协作组

## Abstract

惰性B细胞淋巴瘤（indolent B-cell lymphomas，iBCL）是一组成熟B细胞克隆增殖性疾病，常累及外周血和骨髓。随着第五版世界卫生组织（WHO）造血与淋巴组织肿瘤分类于2022年发布，iBCL的分类方法和命名方式发生了较大变化。本共识将iBCL的疾病谱按照最新版WHO分类标准进行归类，《中国B细胞慢性淋巴增殖性疾病诊断专家共识》正式更名为《惰性B细胞淋巴瘤诊断与鉴别诊断中国专家共识》。中国抗癌协会血液肿瘤专业委员会、中华医学会血液学分会淋巴细胞疾病学组、中国惰性淋巴瘤协作组组织国内相关的血液肿瘤与病理学专家经过多次讨论，对这一共识进行了更新修订，以符合临床实际需求。

## Abstract (en)

Indolent B-cell lymphoma（iBCL）represents a group of mature B-cell clonal proliferative disorders that frequently involve the peripheral blood and bone marrow. In light of the fifth edition of the World Health Organization（WHO）classification of haematolymphoid tumors published in 2022, some changes have been introduced in the classification and nomenclature of certain iBCL subtypes. Accordingly, in this consensus, the disease spectrum of iBCL was reclassified based on the 2022 WHO framework. Reflecting this update, the Chinese expert consensus on the diagnosis of B-cell chronic lymphoproliferative disorders has been formally renamed as expert consensus on the diagnosis and differential diagnosis of indolent B-cell lymphomas in China. This consensus has been revised by the Hematological Oncology Committee of China Anti-Cancer Association, Lymphoid Disease Group, Chinese Society of Hematology, Chinese Medical Association, and Chinese Working Group for indolent lymphoma, following extensive discussions among domestic experts in hematologic oncology and pathology, with the goal of aligning with current clinical practice.

惰性B细胞淋巴瘤（indolent B-cell lymphomas，iBCL）是一组成熟B细胞克隆增殖性疾病，常累及外周血和骨髓，其诊断与鉴别诊断一直是临床工作的难点。自《中国B细胞慢性淋巴增殖性疾病诊断专家共识（2014年版）》[1]和《B细胞慢性淋巴增殖性疾病诊断与鉴别诊断中国专家共识（2018年版）》[2]发布以来，我国医务工作者对iBCL的诊断与鉴别诊断水平有了显著提高。随着第五版世界卫生组织（WHO）造血与淋巴组织肿瘤分类于2022年发布[3]，iBCL的分类方法和命名方式发生了较大变化，有必要对现有共识进行更新。考虑到iBCL疾病谱的连贯性，不再区分是否能通过骨髓或外周血诊断的亚型，而是将iBCL的疾病谱按照2022年第五版WHO分类标准进行归类，故将《中国B细胞慢性淋巴增殖性疾病诊断专家共识》更名为《惰性B细胞淋巴瘤诊断与鉴别诊断中国专家共识》。中国抗癌协会血液肿瘤专业委员会、中华医学会血液学分会淋巴细胞疾病学组、中国惰性淋巴瘤协作组组织国内相关血液肿瘤与病理学专家经过多次讨论，对这一共识进行了更新，以符合临床实际需求。

一、定义

本共识所指iBCL是第五版WHO分类[3]中表现为成熟小B淋巴细胞克隆性增殖及其需要鉴别的亚型，具体类型包括单克隆B淋巴细胞增多症（monoclonal B-cell lymphocytosis，MBL）、慢性淋巴细胞白血病（chronic lymphocytic leukemia，CLL）/小淋巴细胞淋巴瘤（small lymphocytic lymphoma，SLL）、套细胞淋巴瘤（mantle cell lymphoma，MCL）、边缘区淋巴瘤（marginal zone lymphoma，MZL）、毛细胞白血病（hairy cell leukemia，HCL）、伴显著核仁的脾B细胞淋巴瘤/白血病（splenic B-cell lymphoma/leukemia with prominent nucleoli，SBLPN）、脾弥漫性红髓小B细胞淋巴瘤（splenic diffuse red pulp small B-cell lymphoma，SDRPL）、滤泡性淋巴瘤（follicular lymphoma，FL）、淋巴浆细胞淋巴瘤（lymphoplasmacytic lymphoma，LPL）/华氏巨球蛋白血症（Waldenstrm macroglobulinemia，WM）、冷凝集素病（cold agglutinin disease，CAD）以及IgM型意义未明的单克隆丙种球蛋白血症（monoclonal gammopathy of undetermined significance，MGUS），其中，HCL、脾边缘区淋巴瘤（SMZL）、SBLPN、SDRPL又统称为脾脏B细胞淋巴瘤/白血病。此外，还包括部分B细胞慢性淋巴增殖性疾病-不能分类型（B-CLPD-U）。

二、iBCL共同特征[2]

1. 临床特征：中老年发病；临床进展缓慢，多数呈惰性病程；可向侵袭性淋巴瘤转化；治疗后可缓解，但难以治愈。

2. 形态学：以小到中等大小的成熟淋巴细胞为主，部分可见核仁。

3. 克隆性B细胞：确认单克隆性对于iBCL的诊断至关重要，克隆性检测的常用方法包括：①流式细胞术：主要通过检测B细胞sIg轻链限制性表达明确克隆性。克隆性成熟B细胞的免疫表型特征为sIg轻链κ或λ限制性表达和抗原异常表达。当κ/λ>3∶1或<0.3∶1时提示单克隆性。少数病例不表达κ和λ（CD19阳性且sIg阴性细胞>25％），也提示B细胞的单克隆性，必要时应进行IgH/Igκ/Igλ基因重排检测。②遗传学：常规染色体核型分析或荧光原位杂交（FISH）技术检测克隆性染色体异常。③分子生物学：PCR检测IgH、Igκ、Igλ基因重排可判断B细胞存在克隆性异常。

三、各主要iBCL的诊断要点

（一）CLL/SLL

CLL/SLL为最常见的iBCL，以小淋巴细胞在外周血、骨髓、脾脏和淋巴结聚集为特征。中位发病年龄60～75岁，男女比例为2∶1。常表现为外周血淋巴细胞绝对值增多或比例增高[4]。

1. 细胞形态：成熟小淋巴细胞形态，涂抹细胞易见，伴有核仁的幼稚淋巴细胞比例≤15％，若>15％（WHO新诊断标准）则应诊断幼稚淋巴细胞进展型CLL（PP-CLL）。

2. 免疫表型：表达CD19，特征为CD5和CD23与CD19共表达，CD200在CLL中高表达，表达ROR1，但CD20和sIg弱表达，FMC7、CD22和CD79b常阴性或弱表达，不表达cyclin D1（免疫组化，IHC）与CD10。通常表达LEF1（IHC）。

可根据流式细胞术RMH（Royal Marsden Hospital）免疫标志积分与其他iBCL鉴别[5]（表1），4～5分为CLL，0～2分为其他iBCL，积分3分时建议参考CD200、CD43的表达情况进行鉴别，并进行FISH检查除外MCL等[6]。另外，LEF1在CLL中通常阳性，在其他iBCL中少见阳性，有助于鉴别。

**表1:** 慢性淋巴细胞白血病RMH免疫标志积分系统

免疫标志	积分	
	1	0
CD5	阳性	阴性
CD23	阳性	阴性
FMC7	阴性	阳性
slg	弱表达	中等/强表达
CD22/CD79b	弱表达/阴性	中等/强表达

3. 遗传学特点[6]–[7]：免疫球蛋白重链可变区（IGHV）基因突变状态、染色体异常及TP53基因异常是CLL诊断时需要评估的关键遗传学特征。基于IGHV基因突变状态可将患者分为IGHV突变型与IGHV未突变型，前者通常表现为疾病进展缓慢，预后较佳。染色体核型分析或FISH可检测到常见染色体异常包括del（13q14）、del（11q22）（ATM）、+12、del（17p）（TP53）等。除上述染色体异常外，CLL的重现性基因突变主要包括NOTCH1、MYD88、TP53、ATM、SF3B1、FBXW7、POT1、CHD2、RPS15、IKZF3、ZNF292、ZMYM3、ARID1A和PTPN11。具有染色体复杂核型异常、del（17p）和（或）TP53基因突变的患者预后最差，而del（11q）是另一个预后不良标志。

4. 细胞计数：2022年版WHO分型规定，CLL诊断标准之一为外周血单克隆B淋巴细胞≥5×10^9^/L，如果没有髓外病变，单克隆B淋巴细胞<5×10^9^/L时即使存在血细胞减少或疾病相关症状也不诊断为CLL。2018年国际CLL工作组则明确规定，外周血单克隆B淋巴细胞<5×10^9^/L，如存在CLL细胞浸润骨髓所致的血细胞减少时诊断为CLL[8]。国内大多数专家也认为这种情况在排除其他原因导致的血细胞减少后，其临床意义及治疗同CLL，因此应诊断为CLL[4]。

SLL指非白血病患者，具有CLL的组织形态与免疫表型特征，主要累及淋巴结和（或）肝、脾及骨髓，但外周血B淋巴细胞<5×10^9^/L。SLL的诊断应经淋巴结等活检组织病理学检查证实。

推荐对有治疗指征的CLL/SLL患者进行预后相关因素检测，如TP53基因突变/缺失、IGHV突变频率和片段使用、染色体核型，以及与BTK抑制剂敏感性相关的基因，如BTK、PLCG2和BCL2基因突变等。

在CLL/SLL发生发展过程中，可能出现向侵袭性淋巴瘤进展/转化，有两种形式：加速期CLL/SLL和Richter转化（RT）[3]。

（1）加速期CLL/SLL包括两种形式：①组织学侵袭型CLL/SLL：淋巴结增殖中心扩张或融合（>20倍视野）或高增殖指数（Ki-67>40％或每个增殖中心核分裂象>2.4个）；②PP-CLL：外周血幼稚淋巴细胞比例增加（>15％），通常伴有TP53异常，且要除外MCL。

（2）RT：需要淋巴结或其他组织病理进行确诊，通常进展为弥漫大B细胞淋巴瘤，少数进展为经典型霍奇金淋巴瘤或组织/树突状细胞肿瘤。进展为弥漫大B细胞淋巴瘤的患者建议确定克隆来源，若与CLL为同一克隆来源，则预后不佳，若非同一来源，与非转化大B细胞淋巴瘤预后相同[9]。

（二）MBL

指健康个体外周血存在低水平的单克隆B淋巴细胞，并排除CLL/SLL与其他iBCL。免疫分型示B细胞克隆性异常，外周血B淋巴细胞<5×10^9^/L，无肝、脾、淋巴结肿大（所有淋巴结最大直径<1.5 cm）、无贫血及血小板减少、无iBCL的其他临床症状。大多数MBL为CLL表型，但也存在其他表型的MBL。CLL表型的MBL依据外周血克隆性B淋巴细胞计数分为低计数型MBL（<0.5×10^9^/L）和高计数型MBL（≥0.5×10^9^/L）。低计数型MBL很少进展，不需要进行监测。而高计数型MBL的生物学特性与CLL Rai 0期患者类似，应该每年常规随访1次[4]。诊断非CLL表型的MBL需要充分除外其他iBCL侵犯外周血可能。

（三）MCL[10]

MCL占非霍奇金淋巴瘤（NHL）的6％～8％，中位发病年龄60～70岁，男女比例为2～4∶1。多数患者诊断时即处于晚期（Ⅲ/Ⅳ），结外播散常见（消化道、骨髓、外周血）。MCL多呈侵袭性，预后不良。少部分患者在临床中惰性起病，常表现为外周血和骨髓淋巴细胞增多（以成熟小淋巴细胞为主），常有脾大，而无淋巴结肿大。

MCL诊断要点：

1. 免疫表型：同时表达CD5和cyclin D1，CD10、CD23（50％弱阳性）[11]和Bcl-6常阴性。CD20、CD79b和sIg的表达较CLL强，且CD23阴性、CD200阴性、FMC7阳性，可以与CLL相鉴别。免疫组化SOX11阳性。

2. 遗传学特点：t（11;14）（q13;q32）导致CCND1/IGH重排是MCL特征性遗传学改变，一般不见于其他iBCL。对于CCND1重排阴性者，可查CCND2、CCND3重排。90％以上的MCL继发其他遗传学异常，染色体拷贝数异常包括del（11q22）（ATM、BIRC3）、del（9p21）（CDKN2A/B）、del（13q14）（RB1）、del（17p）（TP53）、amp（3q26）（PIK3CA）、amp（11q13）（CCND1），基因突变包括ATM（突变率>40％）、TP53（突变率>25％）、NSD2、KMT2A/C/D、S1PR1、CARD11、SMARCA4、SP140、NOTCH1/2（突变率均<15％）[12]。

3. 组织形态学：MCL组织学常呈弥漫性、结节性和套区型生长模式，细胞形态通常为成熟小淋巴细胞，也可以出现母细胞变异型、多形性型。

主要依据典型的组织形态学特点结合成熟B细胞免疫表型特征，以及CD5和cyclin D1阳性，可诊断MCL。对于白血病型非淋巴结性MCL，如肿瘤细胞免疫表型符合典型MCL、常规染色体核型分析或FISH检出t（11;14）亦可诊断MCL。如果组织形态学特征和免疫表型符合典型MCL，Cyclin D1和t（11;14）均阴性，但SOX11阳性，亦可诊断MCL，有条件单位可以加做FISH检测CCND2或CCND3重排，55％的患者为阳性[13]。具体诊断分型标准请参考《套细胞淋巴瘤诊断与治疗中国指南（2022年版）》[10]

（四）MZL

MZL包括黏膜相关淋巴组织（MALT）结外边缘区淋巴瘤（EMZL）、淋巴结MZL（NMZL）、SMZL、原发性皮肤MZL（PCMZL）以及儿童淋巴结MZL（PNMZL），其中EMZL最常见。以侵犯骨髓或外周血为表现者，SMZL最多，其次为NMZL，PCMZL和PNMZL罕见[3],[14]。

MZL的诊断需要结合形态学、组织病理学、免疫表型、分子遗传学、病原学和发病部位等。各亚型的诊断要点如下[15]。

1. 临床表现：EMZL一般以结外病灶起病，常见于胃、眼附属器、唾液腺、皮肤、肺、乳腺、甲状腺等，可伴引流区域局部淋巴结肿大，多为Ⅰ/Ⅱ期，累及远处淋巴结或骨髓等少见。NMZL以淋巴结肿大为主，可伴骨髓侵犯。SMZL以脾大为主要表现，几乎均伴有骨髓侵犯，白细胞计数升高，可伴脾周淋巴结肿大，远处淋巴结肿大少见。PCMZL一般仅出现皮肤侵犯，淋巴结及其他部位受累少见。PNMZL以青少年男性为主，主要为头颈部淋巴结肿大，预后良好。MZL均较少出现系统性B症状。

2. 组织形态：淋巴细胞小至中等大小，胞质较丰富，核染色质致密，胞体偏大的中心母细胞和免疫母细胞散在可见，但一般不成片出现，伴有浆样分化的细胞在各MZL亚型中均可见。如果是NMZL，淋巴结组织中可见肿瘤细胞围绕滤泡增生，并突入滤泡间区和滤泡（滤泡植入），但仍可检测到残存的滤泡树突细胞网。对于SMZL，肿瘤细胞同时侵犯脾白髓和红髓，以白髓为主，红髓可见小的淋巴细胞结节形成，同时侵犯髓窦和髓索。

3. 免疫表型：通常表达B细胞标志，如CD19、CD20、PAX5等，不表达CD5、CD10、CD103、Cyclin D1、SOX11、LEF1。极少表达生发中心标志（CD10、BCL6、HGAL和LMO2）。IRTA1和MNDA可在EMZL和NMZL中表达[16]–[17]。

4. 遗传学特点：3号和18号染色体三体在所有类型MZL中均较常见，t（11;18）（q21;q21）导致的BIRC3/MALT1易位是EMZL最常见的异常（15％～50％），特别是胃和肺的EMZL。t（14;18）（q32;q21）导致的IGH/MALT1易位在非胃MALT中常见，占15％～20％。7q31-32缺失在SMZL中的发生率高达39％，在其他类型中罕见[15]。

TNFAIP3突变在EMZL中普遍存在，尤其是眼附属器MZL；TBL1XR1和GPR34突变在唾液腺MZL中常见；甲状腺MZL患者常发生TET2基因突变。而在SMZL和NMZL中，KLF2和NOTCH2基因突变常见。PTPRD突变则多见于NMZL[15]。

SMZL有两个确诊方法：①脾脏病理符合SMZL特点，细胞免疫表型在CLL表型积分系统中≤2分；②外周血或骨髓典型的细胞形态学特点+细胞免疫表型+骨髓病理可见CD20阳性肿瘤细胞窦内分布。若无脾脏病理，通过骨髓活检等进行综合诊断也可作为最低诊断标准，但常需要与其他类型iBCL仔细鉴别，有时难以确诊，尤其难以与HCL变异型（HCL-v）和SDRPL准确区分[14],[18]。

（五）HCL[19]

HCL的中位发病年龄为60～70岁，男女比例为5∶1。1/4患者可无症状，多数患者无淋巴结肿大，最突出的特征是脾大和全血细胞减少，白细胞计数很少超过10×10^9^/L，且伴单核细胞减少。诊断要点如下：

1. 免疫表型：表达成熟B细胞相关抗原，且CD20和CD22强阳性。HCL细胞CD11c和CD25强阳性，CD103、CD123、CD200、FMC7和sIg阳性，Annexin A1（IHC）在HCL中特异性表达。CD5、CD10、CD23和CD43阴性。

2. 组织形态：外周血肿瘤细胞周边毛绒状突起。骨髓病理呈“煎鸡蛋”样改变，并常伴纤维化。

3. 基因突变：BRAF V600E突变率高达90％以上。

依据典型的细胞周边毛绒状突起和（或）骨髓病理“煎鸡蛋”样特点，结合免疫表型共表达CD20、CD11c、CD103、CD25和Annexin A1，可诊断HCL。对于BRAF突变阴性患者，多存在IGHV4-34重排，常伴有MAP2K1突变，且临床病程侵袭性更强。

（六）SDRPL

临床较罕见，以脾大为主要表现，在临床与免疫表型上与SMZL无法区分，只能通过脾脏活检鉴别。SMZL脾脏病理通常表现为脾白髓扩张，也可伴有红髓侵犯，而SDRPL相反，表现为脾红髓受累扩张，而白髓萎缩不易辨识。通常CD5、CD10阴性，Cyclin D1和Annexin A1阴性，但70％表达Cyclin D3。此外，CD180在SDRPL中强表达，可作为与HCL和SMZL区分的标志[20]。CCND3突变在SDRPL中常见，而罕见于其他亚型。

（七）伴有显著核仁的脾B细胞淋巴瘤/白血病（SBLPN）

主要包括2016年WHO分类中的HCL-v和非CLL表型的B幼淋巴细胞白血病（B-PLL），以脾大和淋巴细胞升高为主要特点，IGHV片段使用偏好于IGHV4-34[3]。诊断主要依据外周血出现伴显著核仁的幼稚淋巴细胞，通常占比20％～95％，同时不符合SMZL、SDRPL、HCL、MCL和CLL的诊断。

（八）FL

FL是一种较常见的iBCL，来源于淋巴结的生发中心，中位发病年龄60～70岁，20岁以下罕见。多数患者诊断时即处于晚期（Ⅲ/Ⅳ），主要侵犯淋巴结、脾、骨髓和外周血。其免疫表型通常为成熟B细胞和生发中心抗原CD10、Bcl-2（IHC）、Bcl-6（IHC）阳性，部分患者CD23阳性，通常不表达CD5。85％以上有t（14;18）（q32;q21）染色体异常，导致BCL2/IGH易位。主要通过淋巴结病理确诊，首选淋巴结切除活检，且建议取PET-CT代谢高的部位进行活检，以便除外向大B细胞淋巴瘤等转化的可能。此外，需要结合发病年龄、累及部位等确诊特殊亚型，如儿童型FL、十二指肠型FL和原位滤泡B细胞肿瘤（ISFN），具体见《中国滤泡性淋巴瘤诊断与治疗指南（2023年版）》[21]

（九）LPL/WM

LPL/WM是一种浆细胞样淋巴细胞增殖性疾病，典型者由肿瘤性小B细胞、浆样淋巴细胞和浆细胞组成，且不符合其他可能伴浆细胞分化的小B细胞淋巴瘤诊断标准。中位发病年龄60岁左右，常累及骨髓、淋巴结和脾，表现为血细胞减少，淋巴结和脾肿大。大多数患者伴单克隆免疫球蛋白增多，大多数为IgM型。LPL侵犯骨髓伴血清单克隆性IgM丙种球蛋白时诊断为WM。

1. 免疫表型：常分为两群细胞，B细胞表达成熟B细胞相关抗原，与MZL基本相同，可以有CD5或CD23弱表达。浆细胞群CD38和CD138阳性，一般CD19阳性，CD56阴性，与正常浆细胞表型一致，但轻链呈限制性表达。

2. 细胞遗传学：del（6q）见于40％或以上WM患者，MYD88 L265P突变发生率高达90％以上，是相对高发的遗传学异常，具有鉴别诊断价值。

WM诊断要点[22]：

（1）血清中检测到单克隆性IgM（无论数量）；

（2）骨髓中可见浆细胞样或浆细胞分化的小淋巴细胞，呈间质型、小梁旁分布（无论数量）；

（3）免疫表型：CD19^+^，CD20^+^，sIgM^+^，CD5^-/+^，CD10^-^，CD22^+^，CD23^-^，CD25^+^，CD27^+^，FMC7^+^，通常CD38^+^和（或）CD138^+^，而CD103^-^。但10％～20％的患者也可表达CD5、CD10或CD23；

（4）除外其他已知类型的淋巴瘤；

（5）90％以上WM发生MYD88 L265P突变[23]，但MYD88 L265P突变也可见于其他小B细胞淋巴瘤、弥漫大B细胞淋巴瘤等。

（十）CAD[3]

CAD主要表现为寒冷诱导的红细胞凝集所致血管外溶血，常伴脾大，而无明显淋巴结肿大，90％患者可伴有单克隆性IgM，中位发病年龄60岁左右。其免疫表型无特殊标志，通常CD10阴性，约40％可表达CD5，但不表达BCL6、MUM1、CD23和Cyclin D1[24]。

诊断要点如下：①必要条件：慢性溶血；单特异性DAT实验：C3d强阳性；4 °C时冷凝集素滴度≥64；出现单克隆性B淋巴细胞；排除其他恶性疾病和感染性疾病。②推荐条件：单克隆性IgM；MYD88突变阴性；80％患者出现IGHV4-34偏好性使用。

（十一）MGUS

IgM型MGUS多发生于中老年人，随着年龄增大，发病率增高，多为查体发现，无临床症状。骨髓中肿瘤细胞常为CD5^-^CD10^-^的克隆性成熟B淋巴细胞，可伴有MYD88 L265P突变。具有向WM发展的风险，较少发展为IgM型骨髓瘤、CLL或其他淋巴瘤亚型。

WHO诊断标准为：①血清M蛋白<30 g/L；②骨髓克隆性肿瘤细胞<10％；③无贫血、高黏滞血症表现、肝脾肿大、淋巴结肿大和系统性症状等临床表现，但与部分无症状的WM有重叠。依据2023年第11届WM国际研讨会的（IWWM-11）会议共识，将血中出现单克隆IgM（不论数量），骨髓活检无组织侵犯（流式细胞术可以出现低比例克隆性淋巴细胞或浆细胞），无贫血、高黏滞血症表现、肝脾肿大、淋巴结肿大和系统性症状等临床表现者定义为IgM型MGUS，若出现骨髓克隆性肿瘤细胞，则诊断为无症状性WM[25]。

四、iBCL的鉴别诊断

iBCL需要结合临床表现、细胞形态、免疫表型、细胞分子遗传学、组织病理特点等进行综合诊断与鉴别，各类型iBCL的主要特点及鉴别诊断流程可参考图1。

**图1:**
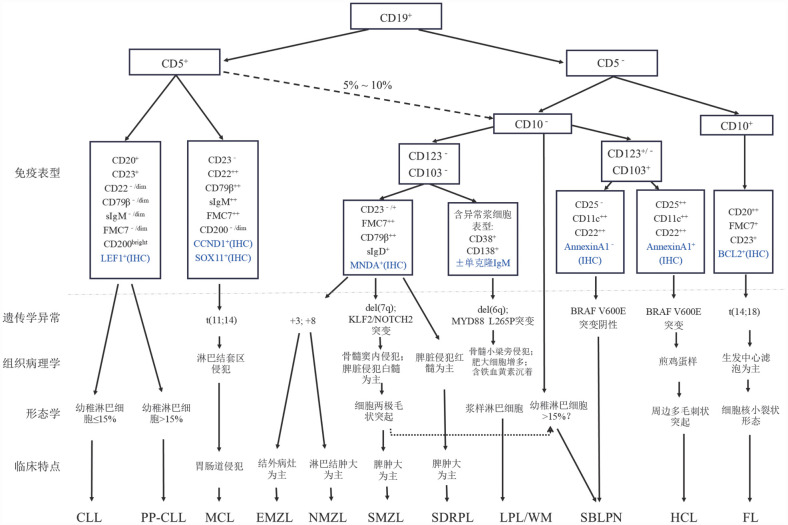
惰性B细胞淋巴瘤鉴别诊断流程图 **注** IHC：免疫组化；CLL：慢性淋巴细胞白血病；PP-CLL：幼稚淋巴细胞进展型CLL；MCL：套细胞淋巴瘤；EMZL：结外边缘区淋巴瘤；NMZL：淋巴结边缘区淋巴瘤；SMZL：脾边缘区淋巴瘤；SDRPL：脾弥漫性红髓小B细胞淋巴瘤；LPL/WM：淋巴浆细胞淋巴瘤/华氏巨球蛋白血症；SBLPN：伴显著核仁的脾B细胞淋巴瘤/白血病；HCL：毛细胞白血病；FL：滤泡性淋巴瘤

1. 细胞形态：iBCL通常表现为成熟小至中等大小淋巴细胞形态，其变异包括以下几种情况：①大细胞形态，需要考虑是否发生大细胞转化（图2A）；②幼稚淋巴细胞样改变，常伴有假核仁，常见于PP-CLL、母细胞变异型MCL、SBLPN（图2B）；③细胞核改变：如呈切迹样改变常见于FL（图2C）；④浆样淋巴细胞，表现为细胞核为淋巴细胞形态、而胞质类似浆细胞形态，常见于LPL和MZL（图2D）；⑤胞膜毛绒状凸起：细胞周边凸起常见于HCL（图2E），而细胞两极毛状凸起，常见于SMZL；⑥篮状细胞，即破坏细胞，无明显细胞核，多为细胞骨架蛋白缺陷所致，多见于CLL，其他iBCL少见（图2F）。

**图2:**
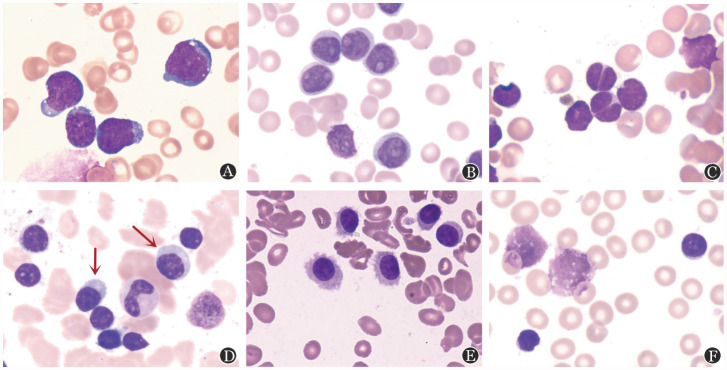
惰性B细胞淋巴瘤主要典型细胞形态（瑞氏-吉姆萨染色，×1 000） **A** 大细胞形态；**B** 幼稚淋巴细胞样改变；**C** 细胞核改变；**D** 浆样淋巴细胞；**E** 胞膜毛绒状凸起；**F** 篮状细胞

2. 骨髓病理学特点：骨髓活检可见间质、结节、窦内或弥漫性浸润特点。CD20阳性细胞窦内浸润对SMZL具有诊断价值。HCL患者骨髓穿刺常为“干抽”，骨髓活检显示间质浸润，呈现为特征性的“煎鸡蛋样”，网状纤维可增加。LPL/WM典型的骨髓活检表现为浆样分化的淋巴细胞呈小梁旁或间质型侵犯，反应性肥大细胞增多和含铁血黄素沉着是其另一个常见表现，有助于与其他惰性淋巴瘤鉴别。

3. 淋巴结或脾脏等组织病理学：对于有浅表淋巴结肿大、易手术切除的患者，除CLL、MCL和HCL可根据典型的免疫表型和细胞遗传学异常确诊而无需手术外，其他类型均建议淋巴结切除，以淋巴结病理学检查作为诊断的主要标准。

脾脏活检是确诊SMZL和SDRPL的金标准，SMZL脾脏病理通常表现为脾白髓扩张，也可伴红髓侵犯，而SDRPL相反，表现为脾红髓侵犯扩张，而白髓萎缩不易辨识，SMZL也可通过骨髓活检进行确诊。

FL的组织学特征是淋巴组织正常结构被破坏，代之以紧密排列、大小和形状相对单一的肿瘤性滤泡，常累及整个淋巴结并浸润至被膜外，伴或不伴局部弥漫性生长，FL的具体分型分级见《中国滤泡性淋巴瘤诊断与治疗指南（2023年版）》[21]。MZL肿瘤细胞围绕滤泡增生，突入滤泡间区，挤压滤泡，但仍可检测到残存的滤泡树突细胞网。LPL/WM患者的淋巴结通常保存滤泡、淋巴窦等结构，但肿瘤性B细胞弥漫浸润，含铁血黄素沉着在LPL/WM中较为常见。

4. 免疫表型：免疫表型是iBCL鉴别诊断的核心，因此，合理的抗体选择与规范的流式细胞术非常关键。除成熟B细胞免疫表型外，关键的鉴别诊断标志包括：CD5、CD10、CD103、CD200、CD11c、CD25、LEF1、Cyclin D1、Annexin A1、MNDA。

（1）CD5阳性一般见于CLL和MCL，5％～15％的MZL和LPL也可表达CD5[26]–[28]，CD5阴性的CLL罕见，5％～15％的MCL可表现为CD5阴性[27],[29]。

（2）CD10阳性主要见于FL，在其他亚型中CD10阳性率低于10％[27]。

（3）CD103阳性主要见于HCL和HCL-v，其他亚型表达率低于10％[27]。

（4）CD200在多种iBCL中表达，其中HCL和CLL表达率和表达强度最高，LPL/WM、FL、MZL均有一定表达率，但表达强度总体低于CLL和HCL，在MCL中表达率最低，仅5％呈弱表达，因此是鉴别CLL和MCL的良好标志[30]。

（5）LEF1在CLL中特异性高表达，见于70％以上的CLL/SLL，而在其他iBCL中表达率低于10％，表达强度较低[31]。LEF1通常需要用免疫组化进行检测。

（6）Cyclin D1主要表达于MCL和HCL，但在HCL中的表达比例和强度明显低于MCL。

（7）MNDA在MZL中表达率高达66％，在LPL中为25％，FL中为5％，可以鉴别MZL与反应性增生，但特异性不高[16]。

5. 遗传学特点：在惰性B-NHL中出现t（11;14）（q13;q32）是MCL的特征性的遗传学异常，推荐进行FISH检测，敏感性为80％～100％。t（14;18）（q32;q21）是FL的主要细胞遗传学异常，由此产生的BCL2/IgH融合基因见于85％～90％的FL患者，FISH检出t（14;18）（q32;q21）是诊断和鉴别诊断的重要依据。+3和+18同时发生在MZL中较为普遍，而在其他惰性B-NHL中少见，不同部位发生的EMZL可出现不同的高发遗传学异常。del（7q31-32）在SMZL中更多见，del（6q）见于40％或以上的WM患者。MYD88 L265P突变在LPL/WM中的发生率高达90％以上，是诊断的重要参考指标，但也并非LPL/WM特有。大多数HCL患者存在BRAF V600E突变，少数不伴BRAF突变的HCL多见于IGHV4-34阳性患者，此时约70％伴有MAP2K1突变。KLF2、NOTCH2基因突变在SMZL和NMZL中常见。这些遗传性异常，除了t（11;14）对MCL具有诊断特异性外，其他均仅作为重要的鉴别诊断参考依据。

五、B-CLPD-U

在临床工作中，10％～15％以骨髓和（或）外周血侵犯起病的iBCL患者的临床特征、细胞形态、免疫表型、细胞/分子遗传学等检查结果不符合上述任何亚型，可诊断为B-CLPD-U[2]。曾有文献将此部分患者归为外周血MZL[14]。但这类患者应尽可能多地获得足够组织标本进行充分诊断，如淋巴结活检、脾切除活检等。这类患者的临床特征及其治疗等有待进一步研究。

六、高级别转化（HGT）[32]–[34]

尽管iBCL表现为惰性病程，进展缓慢，但iBCL均有发生HGT成为侵袭性淋巴瘤的风险，转化可发生在iBCL诊断后或诊断时。确诊HGT的金标准是：①病理组织学的确认，常通过组织活检获得；②克隆相关性，即转化淋巴瘤与惰性淋巴瘤要有相关性。PET-CT在指导活检部位中具有重要作用，且影像高代谢部位与侵袭性淋巴瘤之间具有高相关性，但单独依赖PET-CT不足以诊断HGT。近年来HGT报道较多，包括FL、CLL/SLL、MZL和LPL/WM。临床中，HGT应在患者出现以下情况时予以考虑：新出现的B症状；淋巴结迅速增大或出现新的结外病变部位；乳酸脱氢酶水平升高至大于正常上限2倍；高钙血症。在CLL/SLL患者中，最常见的转化形式是向弥漫大B细胞淋巴瘤的转化；少数情况下也可转化为霍奇金淋巴瘤。MZL和LPL/WM也常转化为弥漫大B细胞淋巴瘤。FL则可能转化为多种组织学类型，最常见的是转化为弥漫大B细胞淋巴瘤，但也可转化为双打击或三打击高级别B细胞淋巴瘤。转化为弥漫大B细胞淋巴瘤的患者预后往往劣于原发弥漫大B细胞淋巴瘤患者。罕见情况下，iBCL可转化为非典型组织学类型，如淋巴母细胞淋巴瘤、浆母细胞淋巴瘤，以及髓系相关恶性肿瘤，如组织细胞肉瘤和树突状细胞肿瘤。惰性淋巴瘤的转化是一个复杂的过程，涉及多种临床表现和组织学类型。HGT的及时识别和准确诊断对于优化患者的治疗策略和改善预后具有重要意义。
